# Oral manifestation in a pediatric patient with pentasomy x. A case report

**DOI:** 10.21142/2523-2754-1101-2023-147

**Published:** 2023-03-26

**Authors:** María Gabriela Acosta de Camargo, Marcia Cancado Figueiredo

**Affiliations:** 1 Departamento de Odontología del Niño y del Adolescente, Universidad de Carabobo. Valencia, Venezuela. macosta@uc.edu.ve Universidad de Carabobo Departamento de Odontología del Niño y del Adolescente Universidad de Carabobo Valencia Venezuela macosta@uc.edu.ve; 2 Facultade de Odontologia da Universidade do Rio Grande do Sul. Porto Alegre, Río Grande del Sur. mcf1958@gmail.com Universidade Federal do Rio Grande do Sul Facultade de Odontologia Universidade do Rio Grande do Sul Porto Alegre, Río Grande del Sur Brazil mcf1958@gmail.com

**Keywords:** pentasomy X, dental enamel, abnormalities, genetic diseases, pentasomía X, esmalte dental, anomalías, enfermedades genéticas

## Abstract

**Aim:**

To describe the oral finding, physical features and medical features with the genetic diagnosis of a Pentasomy X.

**Case report:**

A 6-year-old female patient was referred for oral evaluation presenting acute pain related to ulcers in the attached gingival and dorsum of tongue mucosa. Medical history revealed Pentasomy X associated with interatrial communication, convergent strabismus, recurrent seizures, dysautonomia, episodes of thrombosis and cognitive delay with limited oral communication skills. A palliative oral treatment was applied. Oral hygiene improvement and surveillance were decided considering the absence of dental hypersensitivity, post eruptive enamel breakdowns or dental caries. Less than 30 cases had been reported in the literature, reflecting the rare nature of this genetic disorder and with great difficulties to perform dental treatment. As regards the oral health status of these patients, with an emphasis on dental care, information is totally lacking.

**Conclusion:**

Oral care in patients with genetic syndromes must consider the status in general health to prevent medical complications associated with oral disease or dental treatment. Minimal intervention and surveillance are appropriate options in customized therapy.

## RESUMEN

**Objetivo:**

Describir los hallazgos orales, características físicas y médicas con el diagnóstico genético de una pentasomía X.

**Reporte de caso:**

Paciente de 6 años remitida para evaluación bucal por presentar dolor agudo relacionado con úlceras en la encía adherida y dorso de la lengua. La historia clínica reveló pentasomía X asociada con comunicación interauricular, estrabismo convergente, convulsiones recurrentes, disautonomía, episodios de trombosis y retraso cognitivo con habilidades limitadas de comunicación oral. Se aplicó un tratamiento oral paliativo. Se decidió la mejora de la higiene oral y la vigilancia considerando la ausencia de hipersensibilidad dental, fracturas de esmalte poseruptivas o caries dental. Se han reportado menos de 30 casos en la literatura, lo que refleja la naturaleza rara de este trastorno genético y con grandes dificultades para realizar el tratamiento odontológico. En cuanto al estado de salud bucal de estos pacientes, con énfasis en el cuidado dental, la información es poca.

**Conclusión:**

El cuidado bucal en pacientes con síndromes genéticos debe considerar el estado de salud general para prevenir complicaciones médicas asociadas con enfermedades bucales o tratamientos odontológicos. La intervención mínima y la vigilancia son opciones apropiadas en la terapia personalizada.

## INTRODUCTION

Pentasomy X, also called Penta X-syndrome and Poly-X, is a chromosomal disorder first described by Kesaree and Wooley in 1963^1^. Typically, females have two X-chromosomes; patients with Pentasomy X have three additional X chromosomes. Most polysomies are due to a double nondisjunction in morphogenesis; even though the pathogenesis of pentasomy X is unclear, it is probably caused by successive maternal non-disjunctions. The addition of more than one extra sex chromosome occurs exceptionally and information is generally limited to isolated case reports^2^^,^^3^.

The exact prevalence of pentasomy X is unknown, is a very rare condition that only affects females and the only risk factor is female sex. To our knowledge from 25 to 30 cases have so far been reported, then an approximate incidence of 1 in 85.000 was reported comparing to 49,XXXXY syndrome in male live births. Other numerical abnormalities of the sex chromosomes such as 47,XXX, 47,XXY, 47,XYY and 45,X are relatively common, and occur in approximately 1 of 400 live births^2^^,^^4^^,^^5^.

Pentasomy X significantly impacts systems and organs, facilitating developmental delays. Craniofacial anomalies such as: microcephaly, micrognathia, plagiocephaly, hypertelorism, palpebral fissures, flat nasal bridge, cleft palate and ear malformations have been reported; recurrent seizures in 10-15% of cases and cerebral leukodystrophy, congenital heart defects, kidney dysplasia and musculoskeletal abnormalities have also been noted. Generally, these patients have short stature, and their hands and feet are small with the frequent presence of camptodactyly and clinodactyly. External genitalia are normal, but gonadal dysfunction has been reported. It is also associated with immunoglobulin anomalies and increased susceptibility to infection^2^^,^^3^.

According to buccal findings, few and ambiguous manifestations are reported. Only features mentioned include prominent philtrum, cupid's bow upper lip ^6^, high-arched palate, facial asymmetry ^7^, multiple abnormalities of craniofacial skeleton ^8^, serious dental anomalies ^9^, facial dysmorphisms^4^^,^^10^ or deformed face ^3^.

In the literature, there is not enough information about the dental findings in children with Pentasomy X, even though it is important to know how to diagnosis, manage and guide the patient or parents/careers in this rare disorder. The aim of this case report is to describe the oral finding, physical features and medical features with the genetic diagnosis of a Pentasomy X.

## CASE REPORT

A 6-year-old female patient diagnosed with pentasomy X was referred for oral evaluation presenting acute pain associated with ulcers in the attached gingival and dorsum of tongue mucosa. The mother signed the informed consent. The girl was born from a second gestation of non-consanguineous parents, the mother age was 31-year-old. Parents denied systemic disease or medical conditions. His only brother was diagnosed with an autism spectrum disorder. During pregnancy, four episodes of polyhydramnios required intervention with therapeutic amniocentesis. A Cesarean was performed at 31 weeks. Two weeks later, the patient presented breathing difficulties and received treatment in the Intensive Care Unit for pulmonary atelectasis; and she remained hospitalized for four months. Genetic analyses were requested around that time (see next paragraph). At six months, febrile episodes related to cystic fibrosis were noted, and she was treated with additional oxygen up to 2 years old.

**Figure 1:**
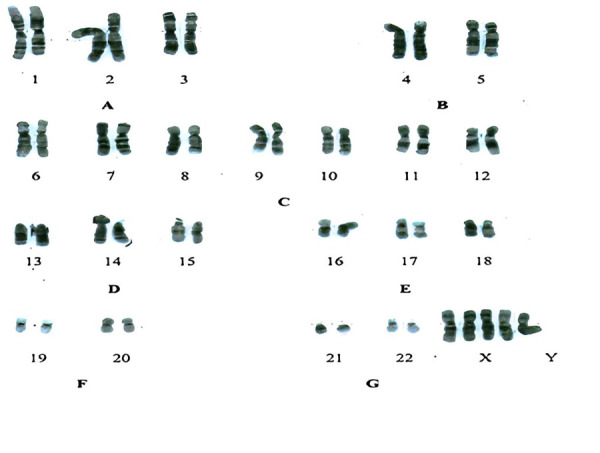
Patient´s kariotype

Chromosome culture was performed from isolated lymphocytes in peripheral blood. Conventional, special high-resolution banding techniques (Trypsin G Bands; C; Q; NOR) were obtained. Autosomal/sexual chromosomal mosaic was ruled out. The conclusion was a karyotype 46, XX; 48, XXXX; 49, XXXXX predominantly XX. The presence of sexual mosaicism was detected XX; XXXX; XXXXX (78; 10: 12%) (Figure 1).

The medical file also revealed recurrent seizures, dysautonomia with low blood pressure, interatrial communication, a few episodes of thrombosis, convergent strabismus, hyperopia, umbilical hernias, and cognitive delay with limited oral communications skills. Diurnal but no nocturnal control of sphincters was reported. Daily medical treatment was 10mg of Nitrazepam and 75mg of Lamotrigine. 

**Figure 2:**
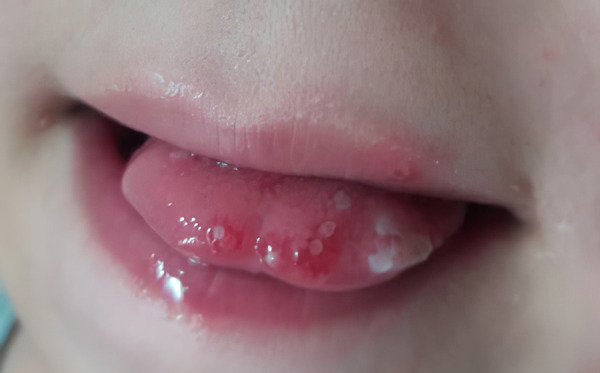
Tongue lesions evaluated by teledentistry

**Figure 3:**
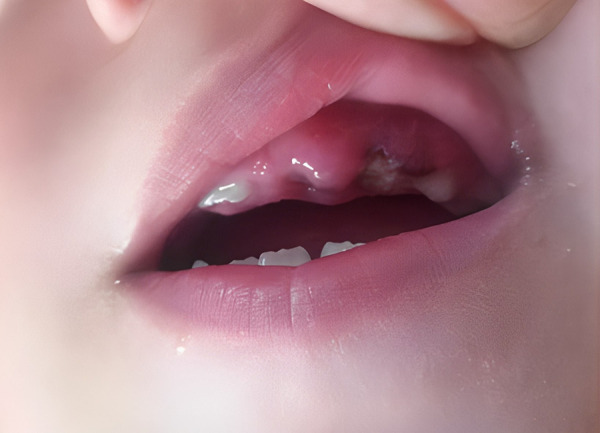
Gingival lesions evaluated by teledentistry

Teledentistry was used for anamnesis and triage of dental urgency, considering COVID-19 pandemic limitations (Figures 2 & 3). Fever and prodromal symptoms were denied. Nutritional limitations were associated with oral pain. Both generated significant concerns. After acute primary herpetic gingivostomatitis was diagnosed, the primary recommendations were adequate oral hygiene, soft and bland diet and palliative treatment with a topical mixture of antacid and diphenhydramine three times per day; before meals to avoid pain and sensitivity. 

**Figure 4:**
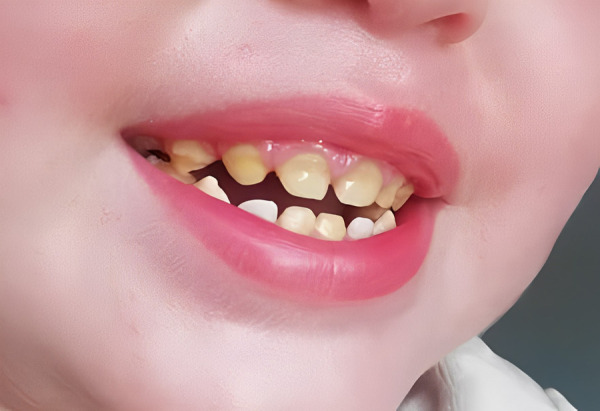
Yellowish primary dentition and open bite

Teledentistry was also used for control follow-up. Complete healing was seen after two weeks. Then, a photo showing an open bite with possibly anterior crossbite and a yellowish primary dentition at three years old (Figure 4). 

**Figure 5:**
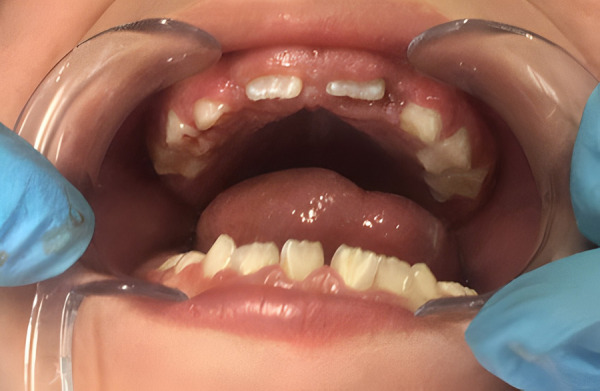
First control visit. Change in primary dentition.

**Figure 6:**
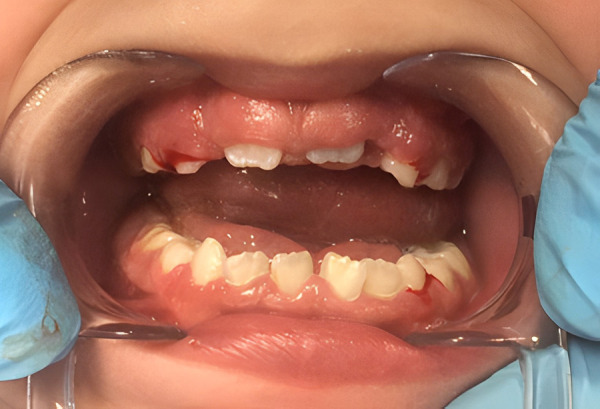
Eruption of permanent incisor with white and creamy demarcated opacities.

Meanwhile, six months later, oral examination revealed brachycephaly, early mixed dentition, acceptable oral hygiene, no dental caries lesions and hyperplastic gingival tissue. Dental enamel was observed in primary dentition without post-eruptive enamel breakdowns or dental hypersensitivity. An improvement in dental coloration in primary dentition was observed in comparison to the above-mentioned photograph at three years of age (Figures 5 & 6). Short stature, adequate weight and difficulties in communications skills were also noted. 

**Figure 7:**
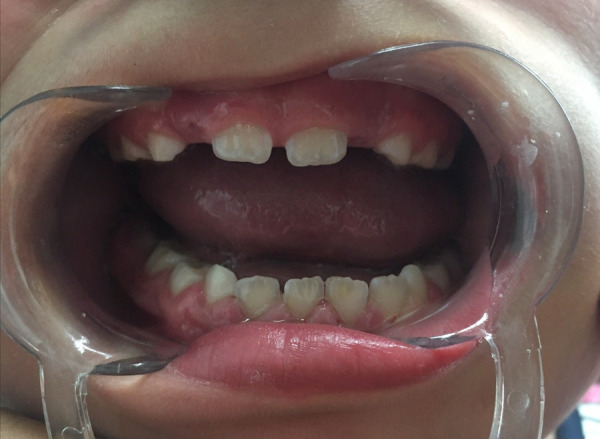
Enamel defects in erupted permanent incisors.

Behavioral problems and interruptive conduct created difficulties during clinical evaluation and dental management. At seven years old, the first permanent molar was absent and upper permanent central and lower central and lateral permanent incisors fully erupted, presenting delayed eruption. The enamel in dental units 11 and 21 showed white and creamy demarcated opacities and 31 showed a yellow demarcated opacity, all of them less than 1/3 of the extent in buccal surfaces, also 41 showed hypoplastic enamel in buccal surface (Figure 7), conversely to symmetrical hypocalcified enamel found in the primary dentition. The gingival tissues showed a hyperplastic appearance. The treatment planning for this enamel defect case was a nonsurgical approach with scaling, oral hygiene instructions considering the absence of dental caries and hypersensitivity, and limitations in the patient´s dental treatment cooperation. Therefore, appointments were scheduled every four months to evaluate gingival tissues and the accumulation of local irritants. 

The patient was evaluated in control visits four times since the first evaluation. The positive outcomes were higher than expected. The mother referred to an improvement in hygiene cooperation and the use of a power-driven toothbrush for oral hygiene. 

## DISCUSSION

The frequency of chromosome abnormalities due to non-disjunction of maternal chromosomes during meiosis is a function of age, with a sharp increase in the slope of the trisomy-age curve between the ages of 30 and 40 years. The basis of this increase, which is a major cause of birth defects, is unknown at present. In recent years, mutations in mitochondrial (mt) DNA have been associated with a growing number of disorders, including those associated with spontaneous deletions of mtDNA (deltamt DNAs). Intriguingly, these pathogenic deltamtDNAs, which are present at extremely high levels in certain patients, are also present at extremely low levels (detectable only by polymerase chain reaction) in normal individuals. The proportion of such deltamtDNAs in normal muscle is a function of age; the shape of this curve is exponential, with the accelerating part of the curve beginning at approximately 30-40 years ^11^. A prevalent hypothesis concerning the cause of the rise in aneuploid conceptions with maternal age is that the changes that accompany normal ovarian aging increase the rate of meiotic errors in the oocyte. Biological aging of the ovary is accompanied by a decline in both the total oocyte pool and the number of antral follicles maturing per cycle, as well as changes in the levels of circulating reproductive hormones. The biological aging hypothesis predicts that aneuploidy rates should be higher in women with a prematurely reduced oocyte pool, and that women with trisomic conceptions should show signs of earlier ovarian aging than women of the same chronological age without trisomic conceptions. Comprehensive studies of aneuploidy in groups of women with known causes of premature ovarian failure remain to be done, though anecdotal evidence does suggest increased rates of pregnancy loss and aneuploidy ^12^. For at least 5% of all clinically recognized human pregnancies, meiotic segregation errors give rise to zygotes with the wrong number of chromosomes. Although most aneuploid fetuses perish in utero, trisomy in liveborns is the leading cause of mental retardation. A large percentage of human trisomies originate from segregation errors during female meiosis I; such errors increase in frequency with maternal age ^13^. In this case report the age of the mother at the time of conception was 31 years, within the range reported as exponential of risk. 

In addition, from the side of odontogenesis, it stands out the human enamel is a highly mineralized tissue whose formation includes cell differentiation, production of an extracellular matrix, regulation of different ions, proteins and pH and other cell functions during different stages of amelogenesis. A plethora of hereditary conditions can generate different enamel defect phenotypes. This is due to the genetic regulation of enamel formation and the expression of thousands of genes in oral epithelium and ameloblasts. Abnormal enamel formation can be associated with an altered protein produced by a transient expression of a gene. Also, many genes associated with abnormal enamel formation can affect other tissues and organs, including the eye, kidney, brain and skin. These findings offer important information on molecular pathways involved in the gene expressions and products in different tissues ^14^^-^^16^. 

Also, inherited enamel defects affect primary and permanent dentition and may present a syndromic form or a non-syndromic form. In pentasomy-X cases, enamel defects have been reported as oral manifestations. In this case, the patient presented primary dentition with yellowish coloration at three years of age, but permanent dentition showed a different phenotype with a significant decrease in severity, extension and clinical presentation, then an inherited enamel defect such as amelogenesis imperfecta was discarded as diagnosis. Considering these findings, different etiological/associated factors (not hereditary) were possibly related to dental condition of the patient. Accordingly, abnormalities in the gestation period and in the first years of life such as premature birth, low birth weight and frequent episodes of fever have been likely associated with enamel developmental defects^16^^,^^17^. Medical history of this patient presents significant systemic risk factors which could be related to failures in amelogenesis in different periods of life affecting both primary and permanent dentition.

Surprisingly, a significant change in dental coloration was observed in primary dentition at three years of age (Figure 4) when compared to clinical evaluation at six years of age, especially in upper primary canines, then spontaneous improvement seemed possible (Figures 5 & 6) and the importance of the clinical examination and limitations in teledentistry are also highlighted. The spontaneous improvement of dental condition has been described in hypomineralized permanent incisors in children with Molar Incisor Hypomineralization ^18^. This enamel defect is defined as a qualitative alteration which affects from one to four first permanent molars with or without the involvement of permanent incisors, characterized by the presence of demarcated opacities, post-eruption breakdowns, atypical dental caries or restorations and delayed eruption ^18^^,^^19^. In this case, permanent incisors presented small demarcated opacities, but the first permanent molars were delayed in eruption. Considering these findings, Molar Incisor Hypomineralization was probable but could not be diagnosed until the first permanent molars are fully erupted. In addition, in Pentasomy-X cases, delayed eruption of permanent teeth has also been reported, and then delayed eruption of first permanent molars observed in this case could be related to the syndrome itself or medication and not necessarily to Molar Incisor Hypomineralization.

Furthermore, high dental caries risk has been reported in children with special health needs. This population represents a heterogeneous group that must be treated using specific caries risk assessment. A recent study showed a difference in dental caries risk between children with congenital heart disease and children with Down syndrome when compared to children without special health needs. A significant difference in caries burden and caries risk between the control group and children with special health needs was also reported. Caries experience in primary dentition was considered a significant predictor of permanent dentition caries incidence^20^. Nevertheless, in this case, even with noted enamel defects and special health needs, no dental caries lesion was detected. Hence, customized surveillance and adequate oral hygiene measures were decided as management. Proper control visits every four months were programmed considering the caries risk assessment. 

On the other hand, the periodontal condition in this Pentasomy-X case may be associated to biofilm accumulation. Medication-induced gingival hyperplasia has been linked to several medications, with a reported prevalence ranging between 0.5% and 85%. Several treatment options have been reported with variable outcomes^20^. The pathogenesis of drug-induced gingival overgrowth is uncertain. Gingival overgrowth induced by drugs such as phenytoin, nifedipine, and cyclosporin develops due to an increase in the connective tissue extracellular matrix. There is an additive effect of those drugs on the degree of gingival overgrowth. Genetic heterogeneity seems to also play an important role in the development of the periodontal disease. Functional difficulties, disfigurement, increased caries, and delayed eruption of permanent teeth are the main complications of drug-induced gingival overgrowth. Discontinuation or change of drug therapy, if it is medically feasible, in addition to surgical and nonsurgical interventions, has been proposed as management approach. Non-surgical measures include scaling, oral hygiene instructions, and antimicrobial mouth rinses. Persistent or relapsed cases had complete resolution with excision of hyperplastic gingiva. Laser-assisted surgeries combined with intensive plaque control measures demonstrated less risk of recurrence^20^. In this case report, gingival hyperplasia was considered mild, and an improvement in oral hygiene using a power-driven toothbrush was achieved.

Strong limitations were faced in this case report related to COVID-19 pandemic restrictions, x-ray study and patient´s cooperation with dental examination and management. Nevertheless, this rare case of Pentasomy-X may provide valuable information to pediatric dentists about the potential of customized therapy and a minimal intervention approach. 

According to Mateus *et al*. until 2010, less than 30 cases had been reported in the literature, reflecting the rare nature of this genetic disorder and with great difficulties to perform dental treatment. As regards the oral health status of these patients, with an emphasis on dental care, information is totally lacking. This is a population that has been little studied and often neglected by health professionals and public figures responsible for creating and implementing policies aimed at this population^6^. The therapeutic success of the treatment must be strongly supported by the cooperation and relationship between patient and parents.

Although the buccal manifestations of this report case are few, brachycephaly, dental retard, enamel defect and gingival overgrowth, they can contribute to assess manifestations in a rare syndrome like pentasomy X in the literature. It is important to highlight there are not pathognomonic buccal signs of the syndrome but are useful for reference in future report cases. Also, this is the only case reported with molecular diagnosis in Venezuela, which contribute to the worldwide prevalence of a rare syndrome. Finally, this case report open research perspective by pointing out that cases like this one could be object of future investigations to report the medical and dental management.
